# Impact of Confinement by COVID-19 in Awake and Sleep Bruxism Reported by Portuguese Dental Students

**DOI:** 10.3390/jcm11206147

**Published:** 2022-10-18

**Authors:** Ricardo Dias, Rui Lima, Ivana Meyer Prado, Anna Colonna, Marco Ferrari, Júnia Maria Serra-Negra, Daniele Manfredini

**Affiliations:** 1 Dentistry Department, Faculty of Medicine, University of Coimbra, 3000-015 Coimbra, Portugal; 2 Department of Pediatric Dentistry, Federal University of Minas Gerais, 6627-Pampulha, Belo Horizonte 31270-901, MG, Brazil; 3 Department of Medical Biotechnologies, School of Dentistry, University of Siena, 53100 Siena, Italy

**Keywords:** awake bruxism, sleep bruxism, COVID-19, mandatory confinement

## Abstract

Confinement by COVID-19 was a stressful period that could potentially trigger awake bruxism (AB) and/or sleep bruxism (SB) behaviors. This study aims to characterize the AB and SB behaviors reported by Portuguese dental students before the pandemic and during the first period of mandatory confinement by COVID-19. Dental students were included in this longitudinal study. They answered the Portuguese validated version of the Oral Behavior Checklist (OBC) before the COVID-19 pandemic emerged (T1) and one month after mandatory confinement started in Portugal (T2). Descriptive statistics and the linear-by-linear association test were performed to assess changes over time (*p* ≤ 0.05). Sixty-four dental students (mean age 22.5 ± 2.8 years; 81.5% females) completed the study protocol. Considering AB, there was a general increase of the behavior from T1 to T2. The percentage of participants who reported to “grind their teeth when waking up” just few times decreased (*p* < 0.001) and the percentage of participants who reported “feeling discomfort/tension in the facial muscles when waking up just few times” increased (*p* = 0.019). Considering SB, there was a significant decrease of the behavior in all samples. The number of “None” report to grinding teeth during sleep or when waking up increased (*p* = 0.012). An increase in the self-reporting of feeling discomfort in masticatory muscles when awake or sleeping was observed (*p* = 0.028). The percentage of participants who did “not remember” any AB or SB activity decreased (*p* < 0.050). The confinement due to COVID-19 resulted in a forced change in dental students’ lifestyles that resulted in an increase of reported AB and a decrease of reported SB. Clinical Significance: In case of confinement periods, students should be encouraged to try normalizing their daily life by creating healthy routines and, by doing so, reducing the possible predisposition to bruxism and its consequences.

## 1. Introduction

Bruxism is defined as a behavior characterized by repetitive masticatory muscle activity during the sleep and/or wakefulness periods in otherwise healthy subjects [1,2]. This muscle activity may result in tooth grinding and tooth contact and bracing or thrusting the mandible without tooth contact. Sleep (SB) and awake bruxism (AB) are distinct entities based on a circadian manifestation, with different etiologies [1,2].

A multifactorial model is involved in the etiology of bruxism, mainly related to central factors rather than peripheral factors, but it must be remarked that specific factors may have different relationships with the different types of masticatory muscle activity [3,4]. While SB features a combination of all bruxism activities (e.g., short- or long-lasting tonic clenching and phasic grinding, with or without teeth contact), AB is commonly characterized by teeth contacting habits or mandible bracing. This means that purported etiological factors may be also different with respect to the circadian manifestations of bruxism. SB is centrally mediated, with a complex interaction of all factors influencing the autonomic system function during sleep. AB is equally centrally mediated, but mostly related to psychosocial factors and behavior changes associated to lifestyle modifications [5,6,7]. 

COVID-19 is caused by SARS-COV2 and represents the causative agent of a potentially fatal disease of great global public health concern [8]. SARS-CoV-2 brought uncertainty to the world population because of suddenly imposed changes in personal, professional, and social habits and routines [8,9]. Lack of faith in the healthcare system to deal with new cases, personal worries about becoming infected, fear of death, increase in hygienic and avoidance behaviors, lack of information, and misinformation fuel excessive fear and create an environment of anxiety and depression that interfere with basic daily activities, including sleep quality [10,11,12].

Extensive measures to reduce person-to-person transmission of COVID-19 have been implemented. One of those was populations’ confinement to try control the community contagion. Quarantines, disruptions of daily life, travel, work, school education, and social isolation that occurred worldwide may impact physical and mental health [11,13]. As people lose social connections, feelings of loneliness and anger may be developing [10,11,12]. 

In Portugal, dental students were forced into a mandatory confinement in the period between 17th of March and 30th April 2020, thus changing their regular study protocols, in most cases returning to their parents’ homes and having to start a domiciliary and an online—on distance period of study. The same happened with all other extra-study and complementary activities. This “new normal” required a personal readaptation and reorganization of each individual, with the inherent psychoemotional implications associated with this forced condition [14,15]. 

It is known that, within their framework of multifactorial etiology, both AB and SB are purportedly associated with periods of emotional tension and alterations in circadian rhythm [2,16,17]. Dental students typically report a high prevalence of bruxism, likely due to the academic pressure inherent to the dentistry course [18,19,20]. Thus, they represent a suitable population to assess the influence of the pandemic and its potential psychological impact on the frequency of bruxism behaviors [14,21,22].

Considering the above premises and based on the null hypothesis that AB and SB neither increases nor decreases in consequence of the mandatory confinement, this study aimed to compare the frequency of AB and SB behaviors reported by Portuguese dental students before the pandemic and during the first period of mandatory confinement by COVID-19.

## 2. Materials and Methods

A longitudinal study was conducted with Portuguese dental students attending the final three academical years, with the initial purpose of making a longitudinal epidemiological characterization of this population in terms of AB and SB prevalence over time. Once the pandemic emerged, the methodology was readapted, and an online form was sent to be answered at home in confinement period (between 10th and 20th of April 2020). The inclusion criteria were being university dental students, age ≥18 years, enrolled in the 3rd, 4th, or 5th year of the course, with good general and dental health (ASA 1 classification), who have never been clinically diagnosed with bruxism. Exclusion criteria were the presence of known and self-reported respiratory or sleep pathology, or the impossibility to answer the questionnaire. All students signed an informed consent form before the start of the study. Participation in the study was voluntary and did not imply any benefit or detriment in academic results. 

The report of AB and SB behaviors was assessed by using the Oral Behavioral Checklist at two different observation points, before and during the pandemic confinement. (T1 and T2), in the morning of a nonspecific business weekday [23]. OBC is a self-report scale for identifying and quantifying the frequency of oral behaviors, developed by Ohrbach and the RDC/TMD Validation Project group, validated to Portuguese (Portugal), and currently considered the best available strategy to collect data on oral behaviors for epidemiological purposes [18,23,24]. For this study, we added the possible answer “Does not know” to the OBC items. Such addition allows avoiding forced negative or positive answers (i.e., “no” or “yes”), when, for instance, the student had no idea of it.

The first session of data collection (T1), before the COVID-19 pandemic, was scheduled between October and November 2019 and taken on site. The second session (T2), four weeks into full mandatory confinement by COVID-19, was organized online in April 2020 (T2). The questionnaire was sent by e-mail and available in the Google Forms platform (Google Inc., Menlo Park, CA, USA). OBC includes items on self-perception of AB behavior over the last 30 days, such as grinding teeth together during waking hours, clenching teeth together during waking hours, or holding the jaw in a rigid or tense position to brace or protect the jaw. To each question, the student had to select the correspondent condition to the period: Does not remember; None; Few Times—less than 1 night per month; Sometimes—1 to 3 nights per month; Most of the Time—1 to 3 nights per week; All the time—4 to 7 nights per week.

All data were analyzed using the Statistical Package for the Social Sciences program for Windows (SPSS Inc., Chicago, IL, USA—Version 26.0). Descriptive statistics were performed to characterize the sample. Linear-by-linear association test and post-hoc Z test with Bonferroni correction were performed [25]. Significance level was set at 5% (*p* ≤ 0.05).

This study was conducted following the principles expressed in the Declaration of Helsinki (revised in World Medical Association, 2013). Ethical approval was waived by the local Ethics Committee of University with protocol ID: 109–CE–2019.

## 3. Results

All students who met the inclusion criteria were invited to participate (87 students). A total of 64 dental students, 17 from the 3rd, 23 from the 4th, and 24 from the 5th years of the dental course, met the inclusion criteria and completed the study protocol (answering the OBC questionnaire before and during COVID-19 mandatory confinement). Most participants were females (81.5), with a mean age of 22.5 (±2.8) years, ranging from 20 to 41 years.

AB—grinding activity (students that do not answer “none” or “does not know”) had a prevalence of 34.5% in T1 and 23.6% in T2, and AB—clenching activity (students that did not answer “none” or “does not remember”) had a prevalence of 79.6% in T1 and 82.9% in T2. AB—bracing/thrusting activity (students that do not answer “none” or “does not know”) prevalence was 34.4% in T1 and 47.0% in T2. SB—grinding activity (students that did not answer “none” or “does not remember”) had a prevalence of 25.1% in T1 and 20.4% in T2. The comparative results of SB and AB activities between T1 and T2 are shown in Table 1 and Table 2, respectively.

Considering AB outcomes, and according to Table 2, there was a significant decrease in the percentage of participants who reported to grind their teeth “a few times” when waking up (12.5% to 4.7%) and who “does not know” it (17,2% to 1.6%) in comparison to an increase of participants who reported “none” (43.7% to 90.6%) (*p* < 0.001). Considering SB outcomes, and according to Table 1, there was a significant increase in individuals reporting “none” who grind their teeth when sleeping (59.4% to 68.6%) and of those who reported it “a few times” (3.1% to 10.9%) and “all the time” (1.6% to 6.3%) in comparison to the decrease of participants who “does not know” (15.6% to 6.3%) (*p* = 0.012). 

There was also a significant increase in participants who reported “a few times” (10.9% to 18.8%) and “most of the time” (9.4% to 12.5%) feeling discomfort in masticatory muscles if waking up at night when compared to the decrease in participants who “does not know” (17.2% to 4.7%) (*p* = 0.028). The same significant increase was observed in the percentage of participants who reported feeling discomfort or tension in the facial muscles when waking up “a few times” (9.4% to 26.1%) and who reported ”none” (42.2% to 52%), when compared to the decrease in the percentage of participants who ”does not know” (10.9% to 0%) this AB activity (*p* = 0.019).

## 4. Discussion

COVID-19 had a strong impact on psychological issues and forced changes in circadian rhythm, with significant reports of moderate-to-severe anxiety, according to the recent literature [11,14]. Following the WHO orientations and public health necessities, restrictions to free movement of people and periods of mandatory confinement of the population to control the COVID-19 pandemic were implemented all around the world, with different schedules and restraints periods over the past two years [26]. Individuals were forced into an extremely stressful and novel living situation, which impacted each subject in different ways and undoubtedly led to psychological consequences [27,28]. Persons modified their natural way of acting, no longer waking up every day to the rhythm of the alarm clock, not getting dressed or washed, not leaving their house to go to university, not taking public or private transport to get there, not interacting for at least 8 h a day with their colleagues, professors, patients, and staff, and no longer organizing the rest of the day to attend to their family, hobbies, pleasure, and social obligations [8]. This unexpected “new normal” created conditions that may reflect in emotion-related muscle activity in vulnerable individuals, thus having a potential impact on AB and/or SB behavior [11,22,29].

To get deeper into this topic, a sample of dental students was used, since they may represent a population of individuals that is typically prone to report AB and SB, possibly in relation with the academic pressure [5,16,20,30,31,32,33,34]. The students taking part to the study experienced first-hand the emotional shock of the first mandatory confinement and experienced a necessary readaptation to a “new normal”.

Applying the OBC questionnaire to the same students at the beginning of the study and during mandatory confinement allowed an evaluation of the changes in bruxism behaviors, viz., how the participants changed their OBC reports after COVID-19-related restraints. With that strategy, the “effect” of emotional stress and lifestyle changes imposed by confinement in the self-recognition of AB and SB by the students was assessed. 

The study hypothesis was that AB and SB increases in consequence of the mandatory confinement. In line with the literature, before the COVID-19 pandemic, a high prevalence of AB and SB was observed [16,18,20,35]. At the data collection point during mandatory confinement, a significant increase in activities of AB and a significant decrease in SB was found. Thus, the study hypothesis was not fully confirmed. 

Regarding AB, the significant increase observed was related to clenching and bracing behaviors, whilst grinding behavior decreased from T1 to T2. This could support the concept that muscle bracing, tooth contact, and clenching behaviors might be the behaviors that should be “typically” associated to AB. These results were in line with the early findings of our group and of Bracci et al. (2018) and confirm the need to change the discriminate between the motor behaviors featuring AB and SB in any future studies [1,2,5,18,35]. 

A recent systematic review found an association between SB and stress symptoms, even if more studies are needed [5,36]. Concerning AB, patients with high levels of stress are almost six times more likely to report awake bruxism [31]. Muscle contraction in AB could be part of the reaction behavior associated with anxiety and stress, thus being enhanced the confinement and uncertainties about the pandemic and the students’ future [37]. On the other hand, SB has a complex generator pattern influenced by several factors that involve nervous system function during sleep [33,37,38]. It can be hypothesized that during confinement, dental students had a more stable circadian rhythm at home with standardized routines and fewer disturbing factors, both contributing to a (re)balance of sleep. Better quality of sleep is usually associated with less disturbance of the autonomic system, less muscle activity, and sleep disturbance [17,38,39]. With an improved sleep, the disruption of sleep architecture that may predispose to SB diminishes [17]. This can explain the fewer reports of SB behaviors during COVID-19 confinement. 

Findings also reported a decrease in the number of participants who answered “does not know” for almost all bruxism activities. On the contrary, the number of patients who answered “Wakes up at night feels discomfort in the masticatory muscles” or “Discomfort or feeling of tension in the facial muscles when waking up” increased. This result highlights the importance of making individuals aware of the differences between AB and SB, as well as what the different activities mean (i.e., bracing, thrusting, clenching, grinding) and the consequences (muscular overload and muscular tenderness) [1]. The decrease in participants who were unaware of the behavior can also justify the increase in all bruxism activities. This also supported the necessity to study bruxism over time and not with single observation point strategies [18,21,40].

This study could serve to adopt preventive measures that can minimize the consequences of increased academic pressure and stress in students’ lives based on the experience of COVID-19 measures. In line with what Lippi et al. (2020) proposed, dental students are encouraged to try normalize their daily life by creating healthy routines, maintaining as far as possible the rhythm that was previously internalized, such as exercising at a specific time each day or maintaining a more or less established schedule [33,34,35]. Despite the confinement, students had to quickly adapt to a new routine of online classes without ever having been absent at any time. In this sense, they never lost the notion of productivity and being busy; this may also explain the reported AB. In a recent work by Colonna et al. (2021) on a general population sample, a similar result concerning the increased report of bruxism during confinement was reported. [41] Our study data are based on two evaluation moments, and are thus not directly comparable to that investigation, with respect. Our results reinforce the importance of evaluating and interpretating bruxism in a same person at multiple observation points. 

This work has the limitation of involving a small sample of dental students that were not representative of the general population. Additionally, the sample consisted mostly of females, who, during forced confinement, might have been culturally predisposed to provide a strong contribution to domestic work. In addition, the potential influence of changes associated with the menstrual cycle, state of humor, sleep disorders, and its implications might be considered [36]. In addition, it must be remarked that this selected sample had access to more information and knowledge about bruxism than laymen people. All of them were physically and emotionally healthy. Since they responded to the same questionnaire a second time, conditioning bias cannot be excluded. However, we cannot neglect that, once a first questionnaire raised attention and awareness to certain specific conditions/behaviors that the student had not considered until that moment, future answers to the same questionnaire may be influenced anyway. More works that take into account for the above drawbacks are essential to evaluate the representativeness of findings about a relationship between emotional stress and bruxism.

## 5. Conclusions

The forced changes in dental students’ lifestyles in consequence of forced confinement due to COVID-19 resulted in an increase in self-reported AB and a decrease in self-reported SB. These findings may suggest that when major forced life changes are introduced, individuals should be encouraged to try normalize their daily life by keeping healthy and steady routines. This might be useful to reduce emotion-related AB and, in turn, reduce the likelihood of clinical consequences. To this purpose, it is also important to make individuals aware of their bruxism behavior to monitor its occurrence.

## Figures and Tables

**Table 1:** Comparative analysis between sleep bruxism reported before the pandemic (T1) and in the first period of mandatory confinement by COVID-19 (T2).

Outcomes	T1 (%)	T2 (%)	*p* *
Grinding the teeth when sleeping (last month)			
Does not know	10 (15.6)	04 (06.3) a	**0.012**
None	38 (59.4)	44 (68.60) b	
Few Times—less than 1 night per month	02 (03.1)	07 (10.9) b	
Sometimes—1 to 3 nights per month	09 (14.1)	04 (06.3) a,b	
Most of the Time—1 to 3 nights per week	04 (06.3)	01 (01.6) a,b	
All the time—4 to 7 nights per week	01 (01.6)	04 (06.3) b	
If wakes up at night feelings a discomfort in the masticatory muscles (last month)			
Does not know	11 (17.2)	03 (04.7) a	**0.028**
None	29 (45.3)	31 (48.4) a,b	
Few Times—less than 1 night per month	07 (10.9)	12 (18.8) b	
Sometimes—1 to 3 nights per month	10 (15.6)	09 (14.1) a,b	
Most of the Time—1 to 3 nights per week	06 (09.4)	08 (12.5) b	
All the time—4 to 7 nights per week	01 (01.6)	01 (01.5) a,b	
You have difficulty opening your mouth when waking up (last month)			
Does not know	07 (10.9)	04 (06.3)	0.523
None	50 (78.4)	45 (70.3)	
Few Times—less than 1 night per month	04 (06.0)	06 (09.4)	
Sometimes—1 to 3 nights per month	02 (03.1)	06 (09.4)	
Most of the Time—1 to 3 nights per week	00 (00.0)	03 (04.6)	
All the time—4 to 7 nights per week	01 (01.6)	00 (00.0)	

T1 = period before the COVID-19 pandemic; T2 = 4 weeks of mandatory confinement by COVID-19; *p* = Probability value. * Linear-by-linear association with post-hoc Z test. Values in bold represent statistical significance. Different letters indicate statistical difference between groups.

**Table 2:** Comparative analysis between awake bruxism reported before the pandemic (T1) and in the first period of mandatory confinement by COVID-19 (T2).

Outcomes	T1 (%)	T2 (%)	*p* *
Grinding the teeth while awake (last month)			
Does not know	03 (04.6)	04 (06.3)	0.116
None	39 (60.9)	49 (76.4)	
Few Times	12 (18.8)	09 (14.1)	
Sometimes	05 (07.8)	01 (01.6)	
Most of the time	04 (06.3)	01 (01.6)	
All the time	01 (01.6)	00 (00.0)	
Clenching the teeth while awake (last month)			
Does not know	02 (03.1)	02 (03.1)	0.444
None	11 (17.3)	09 (14.1)	
Few Times	18 (28.1)	24 (37.5)	
Sometimes	16 (25.0)	14 (21.8)	
Most of the time	15 (23.4)	12 (18.8)	
All the time	02 (03.1)	03 (04.7)	
Bracing the mandible while awake (last month)			
Does not know	08 (12.5)	02 (03.1)	0.260
None	34 (53.1)	31 (48.4)	
Few Times	16 (25.0)	19 (29.7)	
Sometimes	02 (03.1)	05 (07.8)	
Most of the time	03 (04.7)	06 (09.4)	
All the time	01 (01.6)	01 (01.6)	
Grinding the teeth when waking up (last month)			
Does not know	11 (17.2)	01 (01.6) a	**<0.001**
None	28 (43.7)	58 (90.6) b	
Few Times—less than 1 night per month	08 (12.5)	03 (04.7) a	
Sometimes—1 to 3 nights per month	08 (12.5)	02 (03.1) a,b	
Most of the Time—1 to 3 nights per week	06 (09.4)	00 (00.0) a,b	
All the time—4 to 7 nights per week	03 (04.7)	00 (00.0) a,b	
Clenching the teeth when waking up (last month)			
Does not know	13 (20.3)	05 (07.8)	0.387
None	41 (64.1)	32 (50.0)	
Few Times—less than 1 night per month	05 (07.8)	13 (20.3)	
Sometimes—1 to 3 nights per month	02 (03.1)	07 (10.9)	
Most of the Time—1 to 3 nights per week	02 (03.1)	03 (04.7)	
All the time—4 to 7 nights per week	01 (01.6)	04 (06.3)	
Bracing the mandible when waking up (last month)			
Does not know	13 (20.3)	06 (09.4)	0.136
None	42 (65.6)	41 (64.1)	
Few Times—less than 1 night per month	03 (04.7)	09 (14.1)	
Sometimes—1 to 3 nights per month	03 (04.7)	05 (07.7)	
Most of the Time—1 to 3 nights per week	02 (03.1)	01 (01.6)	
All the time—4 to 7 nights per week	01 (01.6)	02 (03.1)	
Discomfort or feeling of tension in the facial muscles when waking up (last month)			
Does not know	07 (10.9)	00 (00.0) a	**0.019**
None	27 (42.2)	34 (52) b	
Few Times—less than 1 night per month	06 (09.4)	17 (26.1) b	
Sometimes—1 to 3 nights per month	15 (23.4)	08 (12.5) a,b	
Most of the Time—1 to 3 nights per week	04 (06.3)	06 (09.4) a,b	
All the time—4 to 7 nights per week	05 (7.8)	00 (00.0) a,b	

T1 = period before the COVID-19 pandemic; T2 = 4 weeks of mandatory confinement by COVID-19; *p* = Probability value. * Linear-by-linear association with post-hoc Z test. Values in bold represent statistical significance. Different letters indicate statistical difference between groups.

## Data Availability

The data that support the findings of this study are available from the corresponding author upon reasonable request.
